# Distribution of lactone-signaling system for expression of secondary metabolite biosynthetic gene clusters in *Streptomyces* species

**DOI:** 10.1042/EBC20250059

**Published:** 2026-08-19

**Authors:** Aiko Teshima, Kuninobu Inada, Kenji Arakawa

**Affiliations:** 1 Program of Biotechnology, Graduate School of Integrated Sciences for Life, Hiroshima University, 1-3-1 Kagamiyama, Higashi-Hiroshima, Hiroshima 739-8530, Japan; 2 Hiroshima Research Center for Healthy Aging (HiHA), Hiroshima University, 1-3-1 Kagamiyama, Higashi-Hiroshima, Hiroshima 739-8530, Japan; 3 Core Facility Management Center, Hiroshima University, 1-4-2 Kagamiyama, Higashi-Hiroshima, Hiroshima 739-8526, Japan

**Keywords:** biosynthesis, receptor, secondary metabolites, signaling molecule, Streptomyces

## Abstract

Many *Streptomyces* species have a signaling-molecule/receptor system for induction of secondary metabolite biosynthetic gene clusters (BGCs). Signaling molecules hitherto discovered and studied contain five-membered heterocycles, and are classified into three groups, including γ-butyrolactones, γ-butenolides, and furans. These molecules except for avenolide-type are biosynthesized by enzymes harboring an AfsA tandem repeat domain. These enzymes (AfsA homologs) catalyze a transfer of β-ketoacyl moiety to the hydroxyl group of dihydroxyacetone phosphate. Alignment of 59 *afsA* homolog genes showed that 86% (51/59) of them located adjacent to their possible signaling-molecule receptor genes, which reminds us to readily predict their signaling-molecule/receptor system for expression of BGCs. Apparent exception is the case of *afsA-arpA* system in *Streptomyces griseus*, whose distance was around 3.91 Mb. Understanding of the signaling-molecule/receptor system may lead to a practical genome mining strategy to awaken silent BGCs through derepression of transcription using the cognate ligands, signaling molecules.

## Introduction

**Figure 1:**
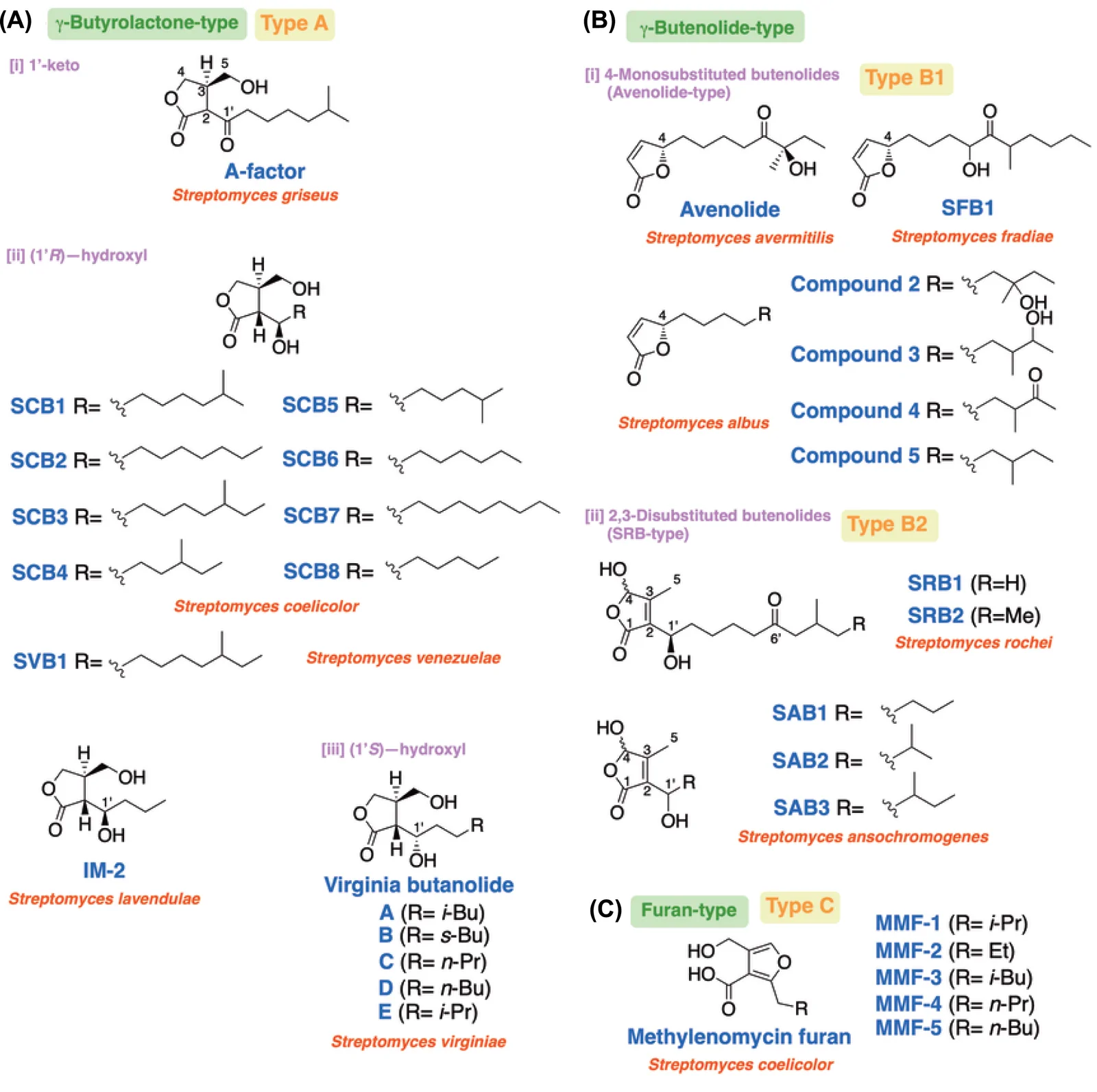
Examples of *Streptomyces* signaling molecules (**A**) γ-butyrolactone-type (Type A), (**B**) γ-butenolide-type (Type B), and (**C**) furan-type (Type C). Me, methyl, *i*-Bu, *iso*-butyl; *s*-Bu, *sec*-butyl; *n*-Pro, *normal*-propyl; *n*-Bu, *normal*-butyl; *i*-Pr, *iso*-propyl; Et, ethyl.

**Figure 2:**
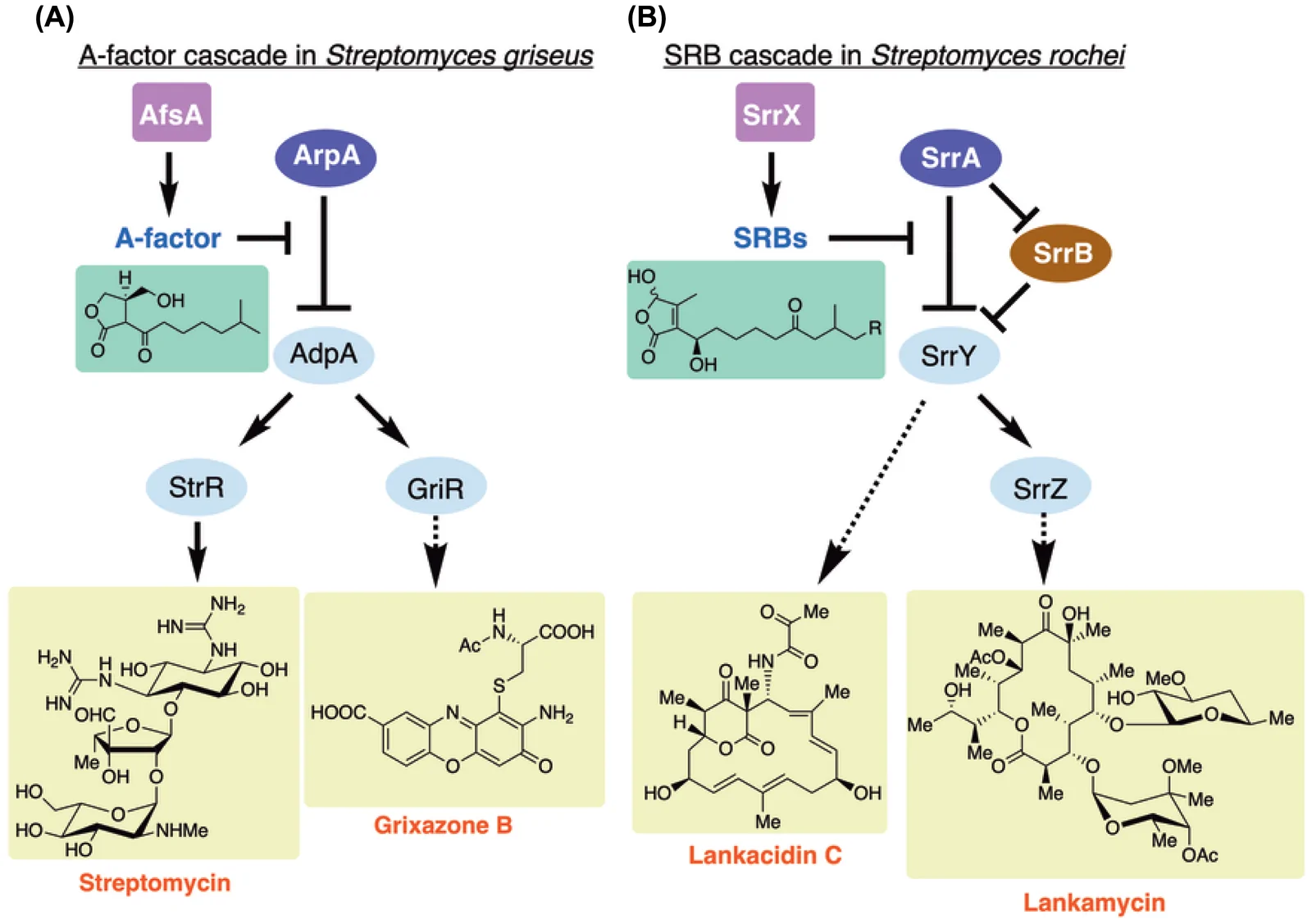
Schematic model of the signaling-molecule/receptor system for expression of BGCs in *Streptomyces* (**A**) A-factor/ArpA system for streptomycin and grixazone production in *S. griseus* and (**B**) SRBs/SrrA system for lankacidin and lankamycin production in *Streptomyces rochei* 7434AN4. Solid arrows indicate direct activation, while dashed arrows indicate indirect activation. ⏊, inhibition.

The filamentous Gram-positive bacterial genus, *Streptomyces*, is well characterized as the prolific producer of secondary metabolites with vast array of significant biological activities [1]. Their biosynthesis is strictly controlled by a diffusible signaling molecule and its cognate receptor [2–8]. *Streptomyces* signaling molecules are often compared with homoserine lactone-type quorum sensors, however, their behaviors are quite different in terms of concentration. Quorum sensing is cell-density dependent phenomena, whereas the *Streptomyces* signaling molecules trigger expression of biosynthetic gene clusters (BGCs) when their concentration reach at a critical concentration (around 10^−9^ M of A-factor in *Streptomyces griseus* [9], 4 nM of avenolide in *Streptomyces avermitilis* [10], and 40–50 nM of SRBs in *Streptomyces rochei* [11]). The well-studied *Streptomyces* signaling molecule is A-factor (Figure 1) for streptomycin production in *S. griseus*. In an early-growth culture, A-factor receptor ArpA tightly binds to a promoter region of its target activator AdpA as shown in Figure 2A. In a mid-stage growth, A-factor is biosynthesized by AfsA protein, and then A-factor reaches at a critical concentration (around 10^−9^ M order), A-factor binds to its specific receptor ArpA. A signaling-molecule/receptor complex, A-factor/ArpA, is then released from the *adpA* promoter region, and then AdpA activates various genes responsible for secondary metabolite biosynthesis, including *strR* and *griR*, as well as genes involved in morphological differentiation [9]. The former gene *strR* activates streptomycin production, while the latter *griR* does grixazone production. Similar signaling regulatory cascade was reported in *S. rochei* 7434AN4 for lankacidin and lankamycin production (Figure 2B). In *S. rochei*, signaling-molecule/receptor complex, SRBs/SrrA, is released from the *srrY* promoter region, and then SARP (*Streptomyces* antibiotic regulatory protein) family transcriptional activator SrrY activates various genes responsible for secondary metabolite biosynthesis [12,13]. The representative regulatory system for expression of BGCs in many *Streptomyces* species is summarized in Table 1.

**Table 1:** Gene sets in the regulatory systems in various *Streptomyces* species^1^

*Streptomyces* strains	Signaling molecule biosynthesis gene	Type of signaling molecules^2^	Signaling molecules	Region number of AfsA homolog gene^3^	Signaling molecule receptor gene	Pseudo-receptor gene	Target activator gene(s) of the signaling molecule/receptor system	Secondary metabolite(s)
*S. coelicolor*	*scbA*	A	SCB1-8	10	*scbR*	*scbR2*	*kasO*	Coelimycin P-1
*S. griseus*	*afsA*	A	A-factor	17	*arpA*		*adpA*	Streptomycin, grixazone
*S. lavendulae*	*farX*	A	IM-2	24	*farA*	*farR2*		Showdomycin
*S. virginiae*	*barX*	A	Virginia butanolides	51	*barA*	*barB*		Virginiamycin
*S. venezuelae*	*jadW1*	A	SVB1	54	*jadR3*	*jadR2*	*jadR1*	Jadomycin
*S. rochei*	*srrX*	B2	SRB1, SRB2	46	*srrA*	*srrB*	*srrY*	Lankacidin, lankamycin
*S. ansochromogenes*	*sabA*	B2	SAB1-3	3	*sabR1*	*sabR2*	*cprC*	Nikkomycin
*S. coelicolor*	*mmfL*	C	MMFs	11	*mmfR*			Methylenomycin
*S. avermitilis*	*aco*	B1	Avenolide	–	*avaR1*	*avaR2*	*aveR*	Avermectin
*S. fradiae*	*orf18*	B1	SFB1	–	*tylP*	*tylQ*	*tylS*	Tylosin
*S. ambofaciens*	N.D	N.D.	N.D.	–	*alpZ*	*alpW*	*alpV*	Kinamycin

N.D., not determined.

**Figure 3:**
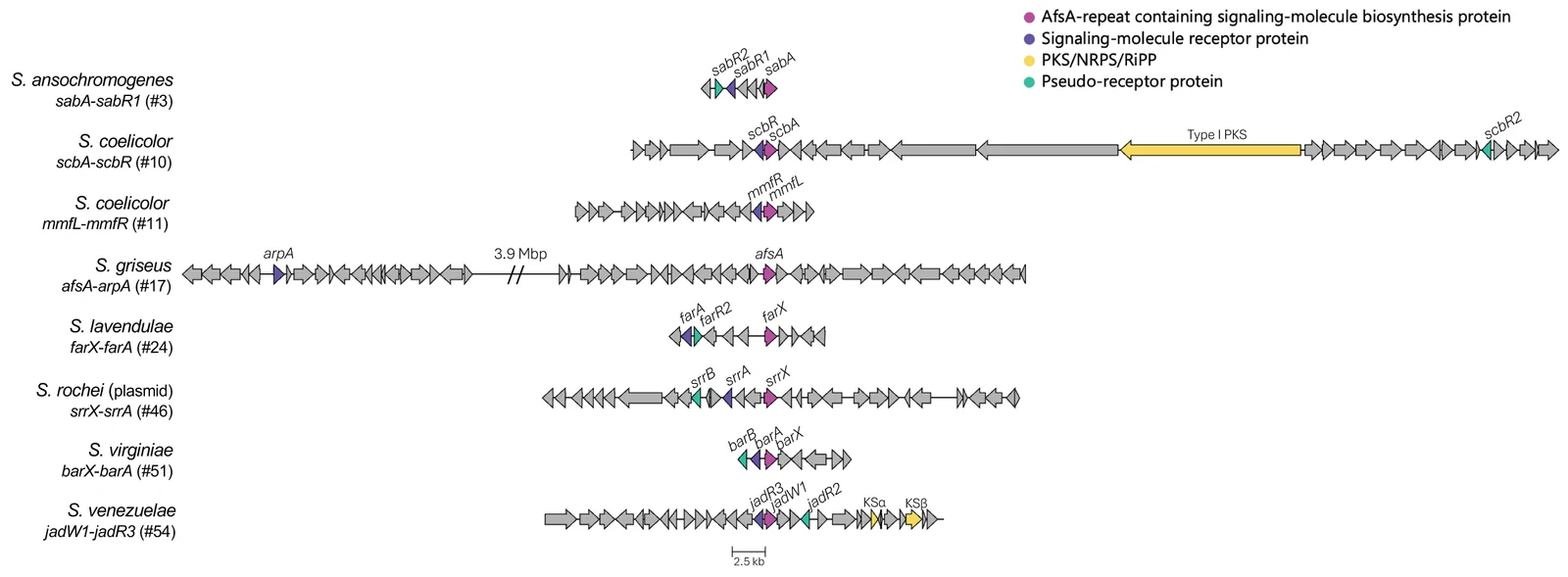
Gene organization of *afsA* homolog and its cognate receptor gene among well-characterized *Streptomyces* strains These genomic regions are listed in Table 1, and controlled by Types A, B2, and C signaling molecules (Figure 1); *scbA*/*scbR* system for coelimycin P1 production in *S*. *coelicolor*, *afsA*/*arpA* for streptomycin and grixazone in *S*. *griseus*, *farX*/*farA* for showdomycin in *S. lavendulae*, *jadW1*/*jadR3* for jadomycin in *S. venezuelae*, *barX*/*barA* for virginiamycin in *S. virginiae*, *sabA*/*sabR1* for nikkomycin in *S. ansochromogenes*, *srrX*/*srrA* for lankacidin and lankamycin in *S*. *rochei*, and *mmfL*/*mmfR* for methylenomycin in *S. coelicolor*.

1 Green highlights indicate the signaling-molecule/receptor system shown in Figure 3.

2 Types A, B2, and C (see Figure 1) are synthesized by AfsA-family enzyme.

3 The cluster number corresponds to Table 2.

We here review the representative signaling-molecule/receptor system and their genetic organization to understand the systematic mechanisms for expression of BGCs in *Streptomyces* species.

## Signaling molecules in *Streptomyces*

### Structural classification of signaling molecules

The diffusible low-molecular-weight signaling molecules induce expression of BGCs at an extreme low concentration (around nM order) in *Streptomyces*. To date, over 12,528 *Streptomyces* genome data (as of 19 February 2026; assembly levels) have been deposited in the GenBank, an NIH genetic sequence database (https://www.ncbi.nlm.nih.gov/genbank/). However, only 40 *Streptomyces and* signaling molecules in 16 strains (0.13% of sequenced strains) has been structure elucidated due to their extremely low yield [2–8]. Recently, Kudo et al. developed resin-assisted isolation strategy and chemoenzymatic method for the synthesis of butyrolactone standards, and identified butyrolactones in nearly half of 31 actinomycete strains across 10 genera [14]. The representative *Streptomyces* signaling molecules hitherto discovered are classified into three groups; (1) γ-butyrolactone-type (Type A), (2) γ-butenolide-type (Type B), and (3) furan-type (Type C) (Figure 1). The most-studied γ-butyrolactone-type molecules contain the 2,3-disubstituted γ-butyrolactone skeletons (Type A; Figure 1A), and they are further divided into three subclasses based on their oxidation degree and C-1’ stereochemistry. The 1’-keto subclass [i] contains A-factor in *S. griseus* [15,16]. Second subclass, the (1’*R*)-hydroxyl subclass [ii], contains SCB1-8 in *Streptomyces coelicolor* [17–19], SVB1 in *Streptomyces venezuelae* [20], and IM-2 in *Streptomyces lavendulae* [21]. Third subclass, the (1’*S*)-hydroxyl subclass [iii], contains virginia butanolides A–E in *Streptomyces virginiae* [22]. The γ-butyrolactone-type share a dominant class (about 60%) of signaling molecules to induce antibiotic production [16,23]. Another two types of signaling molecules harboring five-membered heterocycle rings with four carbons and one oxygen were discovered and classified as γ-butenolide-type (Type B; Figure 1B) and furan-type (Type C; Figure 1C). The γ-butenolide-type molecules (Figure 1B) are further divided into two subclasses, 4-monosubstituted (Type B1; Figure 1B-i) and 2,3-disubstituted butenolides (Type B2; Figure 1B-ii). A most-studied 4-monosubstituted butenolide, named avenolide, induces avermectin production in *Streptomyces avermitilis* at 4 nM concentration [10]. SFB1 acts as an inducer for tylosin production in *Streptomyces fradiae* [24]. Four additional avenolide-like butenolides (compounds 2–5) were isolated from *Streptomyces albus* J1074, although their function in *S. albus* has not yet been elucidated [25,26]. Two 2,3-disubstituted butenolide molecules named SRB1 and SRB2 (termed as ‘SRB-type butanolide’) induce the production of two antibiotics, lankacidin and lankamycin, in *Streptomyces rochei* at 40–50 nM concentration [11,27]. Three additional SRB-type butenolides SAB1-3 were discovered as nikkomycin inducers in *Streptomyces ansochromogenes* [28]. Regarding Type C signaling molecules, five furan-type signaling molecules named methylenomycin furan (MMF) 1–5 (Figure 1C) act as inducers for methylenomycin production in *S. coelicolor* [29].

### Biosynthetic enzyme of signaling molecules

The signaling molecule biosynthetic proteins for γ-butyrolactone-type (Type A), 2,3-disubstituted butenolide(SRB)-type (Type B2), and furan-type (Type C) contain AfsA tandem repeat domains (Pfam03756) [5,30]. On the other hand, 4-monosubstituted butenolide(avenolide)-type (Type B1) could be synthesized by an acyl-CoA oxidase [10]. Hirata *et al.* reported the sequence alignments of the randomly selected 122 AfsA-homolog biosynthetic enzymes for Types A, B2, and C signaling molecules [31]. Eight AfsA homolog enzymes for hitherto identified signaling molecules (Figure 1A,B-ii,C), including five γ-butyrolactones synthetic enzymes (ScbA, AfsA, JadW1, FarX, and BarX), two butenolide molecules synthetic enzymes (SrrX and SabA), and one furan-type molecule biosynthetic enzyme (MmfL) were located in diversed 8 clades [31]. Remarkably, SabA protein for SAB1-3 from *S. ansochromogene*s has only 42% identity (122 aa in 285 aa) and 77% similarity (222 aa in 285 aa) to SrrX protein for SRB1 and SRB2 from *S. rochei*, although they produce SRB-type butenolides. Nevertheless ScbA protein for SCB1-8 in *S. coelicolor* and JadW1 protein for SVB1 in *S. venezuelae* are able to produce same structure of signaling molecule, SCB3 (for *S. coelicolor*) and SVB1 (for *S. venezuelae*) [20], ScbA protein shows 45% identity and 83% similarity to JadW1 protein. Thus, prediction for the type of signaling molecules is a less plausible way from the amino acid sequence homology in biosynthetic enzymes.

## Classification of signaling molecule/receptor system in various *Streptomyces* species

### The signaling molecule/receptor system in *Streptomyces*

The signaling molecule-dependent regulatory pathways in *Streptomyces* lead to induce expression of BGCs, and typically contain the following gene sets; signaling-molecule biosynthesis gene, signaling-molecule receptor gene, target gene of receptor, and pseudo-receptor gene. Hitherto characterized regulatory gene sets for expression of BGCs are summarized in Table 1. The followings are controlled by Types A, B2, and C signaling molecules; *scbA*/*scbR* system controlled by SCBs for coelimycin P1 production in *S*. *coelicolor* [17,18,32–34], *afsA*/*arpA* by A-factor for streptomycin and grixazone in *S*. *griseus* [16,35,36], *farX*/*farA* by IM-2 for showdomycin in *S. lavendulae* [21,37], *jadW1*/*jadR3* by SVB1 for jadomycin in *S. venezuelae* [20,38–41], *barX*/*barA* by virginia butanolides for virginiamycin in *S. virginiae* [22,42–44], *sabA*/*sabR1* by SABs for nikkomycin in *S. ansochromogenes* [28,45], and *srrX*/*srrA* by SRBs for lankacidin and lankamycin in *S*. *rochei* [7,11–13,46–49], and *mmfL*/*mmfR* by MMFs for methylenomycin in *S. coelicolor* [29,50]. Other signaling molecule/receptor system governed by two 4-monosubstituted butenolides (Type B1), avenolide [10,51,52] and SFB1 [53–55], are also shown in Table 1. The regulatory pathway for kinamycin production in *S*. *ambofaciens* is also extensively studied, nevertheless the signaling molecule itself and its biosynthetic gene have not yet been determined [56–59].

### Gene organization of signaling molecule/receptor system

The signaling molecule receptor proteins belong to TetR-family transcriptional regulators harboring a helix-turn-helix DNA-binding motif at the *N*-terminus and a ligand (signaling molecule) binding residues at the *C*-terminus [60,61]. The signaling molecule receptor genes usually locate neighbor to or near the signaling-molecule biosynthetic genes [62], which constitute ‘regulatory-gene-island’ in *Streptomyces* genome. Hence, it is easy to detect the presence of signaling-molecule/receptor systems in *Streptomyces* species [6]. To confirm their vicinal location in the genome, we aligned eight well-studied *afsA* homolog genes (listed in Table 1) and their boundary locus in Figure 3. In this mini-review, we excluded 4-monosubstituted butenolides (Type B2) since their biosynthetic enzymes are acyl-CoA oxidase, not AfsA homolog. As shown in Figure 3, seven *afsA* homologs (*scbA*, *farX*, *jadW1*, *barX*, *sabA*, *srrX*, and *mmfL*) located vicinal position to their cognate receptor homologs (*scbR*, *farA*, *jadW3*, *barA*, *sabR1*, *srrA*, and *mmfR*, respectively) in regions 10, 24, 54, 51, 3, 46, and 11, respectively. On the other hand, a receptor gene *arpA* in *S. griseus* locates at 3.91 Mb away from *afsA* gene (Figure 3; region 17).

**Figure 4:**
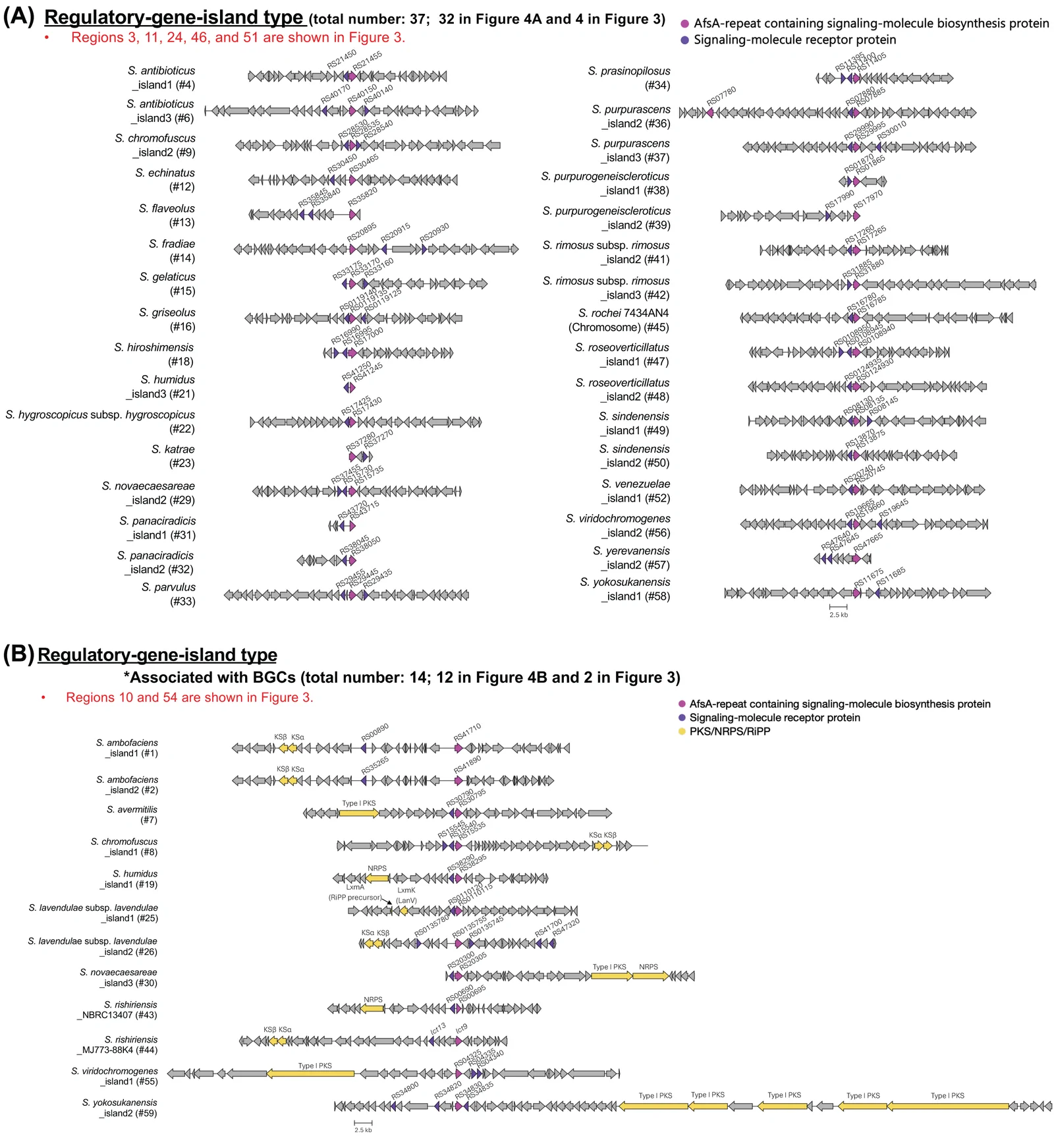
Gene organization of *afsA* homolog and its cognate receptor gene among 60 regions of *Streptomyces* strains (**A**) **Regulatory-gene-island type.** These genomic regions are listed in Table 2, and controlled by Types A, B2, and C signaling molecules (Figure 1). Regions 3, 11, 24, 46, and 51 are shown in Figure 3. (**B**) **Regulatory-gene-island type (associated with certain secondary metabolite BGCs).** These genomic regions are listed in Table 2, and controlled by Types A, B2, and C signaling molecules (Figure 1). Regions 10 and 54 are shown in Figure 3.

**Figure 5:**
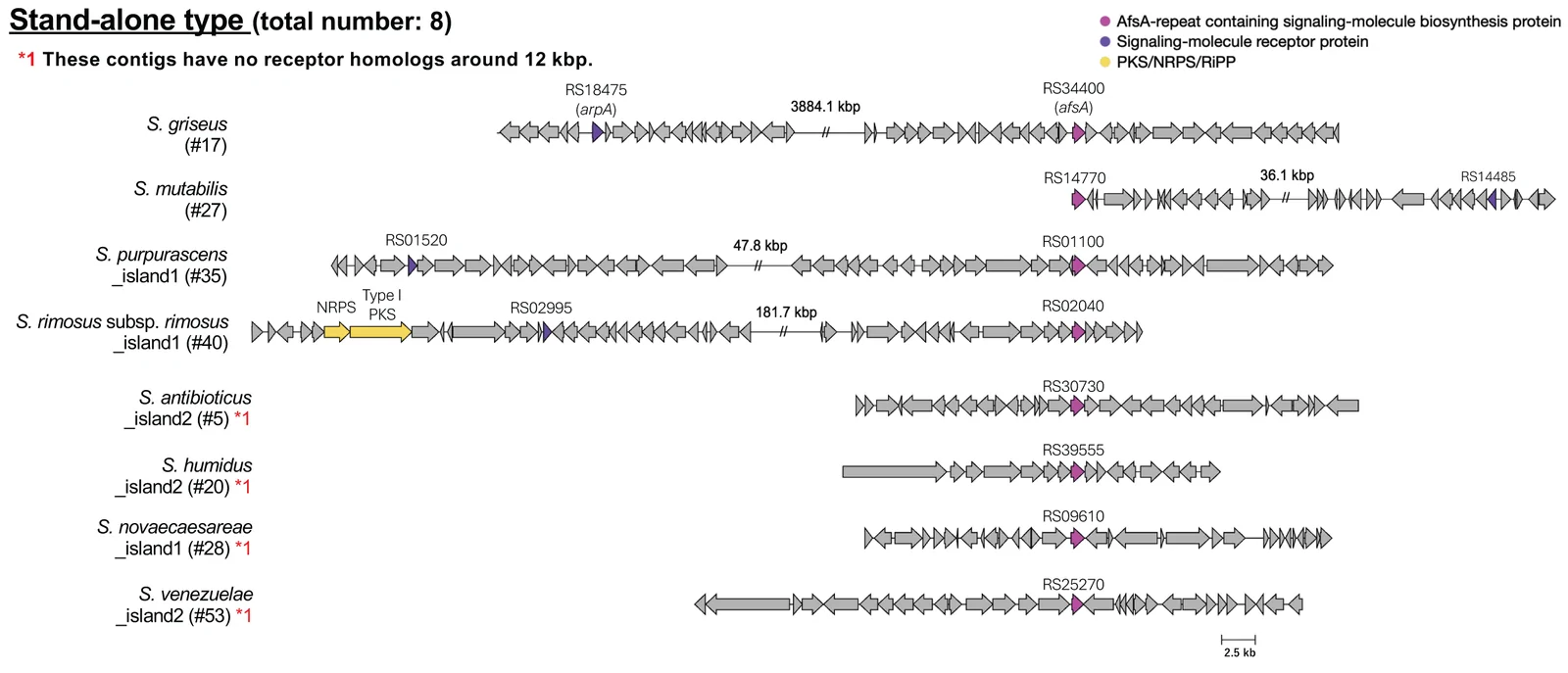
Gene organization of *afsA* homolog and its cognate receptor gene among 60 regions of *Streptomyces* strains; stand-alone type These genomic regions are listed in Table 2, and controlled by Types A, B2, and C signaling molecules (Figure 1).

We further investigated the location of *afsA* homologous genes and their receptor homologs in 59 regions containing *afsA* homologs (Table 2). These *afsA* homologs were previously listed in a preceding literature [31], and the gene regions vicinal to the *afsA* locus were investigated in GenBank database. Around 86% (51/59) are classified into regulatory-gene-island type (Figure 4), while other sets (8/59) are classified into stand-alone type (Figure 5).

**Table 2:** List of the regions harboring AfsA-repeat containing signaling-molecule biosynthesis genes

Region	Strain Name_island No.	Region accession No.	AfsA-repeat containing signaling-molecule biosynthesis protein	
			Protein accession No.	Locus tag
1	*S. ambofaciens* ATCC 23877_island1	NZ_CP012382.1	WP_053125931.1	SAM23877_RS41710
2	*S. ambofaciens* ATCC 23877_island2		WP_053141946.1	SAM23877_RS41890
3	*S. ansochromogenes* 7100	KF170348.1	WP_190059139.1	SabA
4	*S. antibioticus* DSM 40234_island1	NZ_KQ948426.1	WP_107120406.1	AQJ23_RS21455
5	*S. antibioticus* DSM 40234_island2	NZ_KQ948429.1	WP_059195973.1	AQJ23_RS30730
6	*S. antibioticus* DSM 40234_island3	NZ_KQ948433.1	WP_059197793.1	AQJ23_RS40150
7	*S. avermitilis* MA-4680 = NBRC 14893	NZ_BAVY01000028.1	WP_202497367.1	QZ78_RS30795
8	*S. chromofuscus* DSM 40273_island1	NZ_CP063374.1	WP_228040481.1	IPT68_RS15535
9	*S. chromofuscus* DSM 40273_island2	NZ_CP063374.1	WP_308438778.1	IPT68_RS28535
10	*S. coelicolor* A3(2)	NC_003888.3	WP_003972659.1	ScbA (SC_RS33580)
11	*S. coelicolor* A3(2)	NC_003903.1	WP_011039545.1	MmfL (SC_RS01290)
12	*S. echinatus* CECT 3313	NZ_JACHJK010000013.1	WP_225817690.1	FHS34_RS30465
13	*S. flaveolus* JCM 4032	NZ_BMRV01000031.1	WP_189234510.1	IEZ05_RS35820
14	*S. fradiae* ATCC 10745 = DSM 40063	NZ_CP023696.1	WP_031128615.1	BG846_RS20895
15	*S. gelaticus* JCM 4376	NZ_BMTF01000043.1	WP_229867447.1	IE107_RS33170
16	*S. griseolus* NRRL B-2925	NZ_JOFC01000018.1	WP_051422317.1	IH14_RS0119135
17	*S. griseus* IFO13350	NC_010572.1	WP_003971211.1	SGR_RS34400
18	*S. hiroshimensis* JCM4586	NZ_BMUT01000007.1	WP_190022609.1	IE241_RS17000
19	*S. humidus* JCM 4386_island1	NZ_BMTL01000053.1	WP_190154069.1	IE239_RS38295
20	*S. humidus* JCM 4386_island2	NZ_BMTL01000062.1	WP_190154286.1	IE239_RS39555
21	*S. humidus* JCM 4386_island3	NZ_BMTL01000112.1	WP_190154574.1	IE239_RS41245
22	*S. hygroscopicus* subsp. *hygroscopicus* NBRC 13472	NZ_BBOX01000153.1	WP_060945341.1	AXW62_RS17430
23	*S. katrae* NRRL ISP-5550	NZ_JZWV01001363.1	WP_052714006.1	VR44_RS37280
24	*S. lavendulae* FRI-5	AB434932.1	BAG74715.1	FarX
25	*S. lavendulae* subsp. *lavendulae* NRRL B-2774_island1	NZ_JOEW01000009.1	WP_051840626.1	IF07_RS0110115
26	*S. lavendulae* subsp. *lavendulae* NRRL B-2774_island2	NZ_JOEW01000059.1	WP_031943727.1	IF07_RS0135755
27	*S. mutabilis* JCM 4400	NZ_BMTT01000005.1	WP_191886169.1	IE142_RS14770
28	*S. novaecaesareae* NRRL B-1267_island1	NZ_JNWQ01000019.1	WP_051829899.1	OP86_RS09610
29	*S. novaecaesareae* NRRL B-1267_island2	NZ_JNWQ01000035.1	WP_051830126.1	OP86_RS15735
30	*S. novaecaesareae* NRRL B-1267_island3	NZ_JNWQ01000052.1	WP_078588748.1	OP86_RS20305
31	*S. panaciradicis* NBRC 109811_island1	NZ_JAMGBF010000284.1	WP_249841715.1	M8J74_RS43715
32	*S. panaciradicis* NBRC 109811_island2	NZ_JAMGBF010000171.1	WP_249840804.1	M8J74_RS38050
33	*S. parvulus* JCM 4068	NZ_BMRX01000017.1	WP_064732196.1	IE108_RS29445
34	*S. prasinopilosus* NRRL B-2711	NZ_LIRG01000239.1	WP_055694258.1	AOK18_RS11405
35	*S. purpurascens* JCM 4509_island1	NZ_BMUK01000001.1	WP_229872786.1	IE276_RS01100
36	*S. purpurascens* JCM 4509_island2	NZ_BMUK01000003.1	WP_189722829.1	IE276_RS07780
			WP_189722846.1	IE276_RS07885
37	*S. purpurascens* JCM 4509_island3	NZ_BMUK01000012.1	WP_189727138.1	IE276_RS29995
38	*S. purpurogeneiscleroticus* DSM 43156_island1	NZ_NHSG01000062.1	WP_223731419.1	CCS38_RS01865
39	*S. purpurogeneiscleroticus* DSM 43156_island2	NZ_NHSG01000280.1	WP_223734306.1	CCS38_RS17970
40	*S. rimosus* subsp. *rimosus* ATCC 10970_island1	NZ_CP048261.1	WP_003984549.1	SRIM_RS02040
41	*S. rimosus* subsp. *rimosus* ATCC 10970_island2		WP_003979781.1	SRIM_RS17265
42	*S. rimosus* subsp. *rimosus* ATCC 10970_island3		WP_003981106.1	SRIM_RS31880
43	*S. rishiriensis* NBRC 13407	NZ_BEWA01000001.1	WP_281261875.1	Sri02f_RS00695
44	*S. rishiriensis* MJ773-88K4	EU147298.1	ABX71092.1	Lct9
45	*S. rochei* 7434AN4 (chromosome)	NZ_AP018517.1	WP_037663082.1	FYM93_RS16785
46	*S. rochei* 7434AN4 (plasmid pSLA2-L)	AB088224.1	BAC76543.1	SrrX
47	*S. roseoverticillatus* NRRL B-3500_island1	NZ_JOFL01000006.1	WP_030365741.1	IF61_RS0108940
48	*S. roseoverticillatus* NRRL B-3500_island2	NZ_JOFL01000022.1	WP_030368766.1	IF61_RS0124930
49	*S. sindenensis* JCM4164_island1	NZ_BMSG01000004.1	WP_189520203.1	IE145_RS08135
50	*S. sindenensis* JCM4164_island2	NZ_BMSG01000010.1	WP_229825536.1	IE145_RS13875
51	*S. virginiae* MAFF 10-06014	NZ_CP107872.1	BAA23611.1	BarX
52	*S. venezuelae* ATCC 10712_island1	NC_018750.1	WP_158506265.1	SVEN_RS20745
53	*S. venezuelae* ATCC 10712_island2	NC_018750.1	WP_234104285.1	SVEN_RS25270
54	*S. venezuelae* ATCC 10712	NC_018750.1	WP_015037150.1	JadW1 (SVEN_RS29615)
55	*S. viridochromogenes* DSM 40736_island1	NZ_GG657757.1	WP_039931924.1	SSQG_RS04325
56	*S. viridochromogenes* DSM 40736_island2	NZ_GG657757.1	WP_003991561.1	SSQG_RS19660
57	*S. yerevanensis* NRRL B-16943	NZ_JNWJ01000125.1	WP_245685735.1	OP79_RS47665
58	*S. yokosukanensis* DSM 40224_island1	NZ_KQ948209.1	WP_067121117.1	AQI95_RS11675
59	*S. yokosukanensis* DSM 40224_island2	NZ_KQ948223.1	WP_067133519.1	AQI95_RS34830

1 Green highlights indicate well-studied AfsA-repeat containing enzymes in *Streptomyces* (see Table 1 and Figure 3).

In the case of regulatory-gene-island type (Figure 4), 51 signaling-molecule receptor homologs are located at the vicinal position to the *afsA* homolog gene. Among them, some regulatory-gene-island regions (14/51) are located close to or within the potential secondary metabolite BGCs (Figure 4B). For example, *Streptomyces rishiriensis* MJ773-88K4 (region 44) harbors a gene set of signaling-molecule biosynthesis gene *lct9*/receptor homolog gene *lct13* in the lactonamycin biosynthetic gene (*lct*) cluster for type-II polyketide synthase product [63]. It is interesting that region 36 (island #2 in *S. purpurascens* JCM 4509) harbors two copies of *afsA* homologs at a 20.4-kb distance (Figure 4A and Table 2). Their amino acid identity and similarity are 34% and 45%, respectively.

Although many signaling-molecule receptor genes are located near the *afsA* homolog gene, their exact function is not yet clarified except gene sets shown in Table 1 and Figure 3. ‘Functional’ signaling-molecule receptor genes should be confirmed by their mutational analysis and a ligand (signaling molecule) binding assay using their signaling molecules. Double mutation of *afsA* homolog and their possible receptor homolog gene may allow us to predict the function of signaling-molecule receptors in their strains; for example, production of metabolites indicates the involvement of signaling-molecule/receptor system for expression of BGCs, while abolishment of metabolites indicates that the target receptor homolog is not a true signaling-molecule receptor and independent to signaling-molecule/receptor system [46]. This double-mutation strategy will provide us a chance to discover new type of signaling molecules in *Streptomyces*.

Remarkably, the most-studied signaling-molecule/receptor system, A-factor/ArpA in *S. griseus*, is classified into stand-alone type (Figures 3 and 5; region 17). Their genetic locus aparts from 3.91-Mb [AfsA; SGR_6889 (RS34400) at nt_8273444..8274388, ArpA; SGR_3731 (RS18475) at nt_4358770..4359600] in the *S. griseus* chromosome, hence, it is difficult to predict the functional receptor genes on the basis of genetic locus of signaling-molecule/receptor system. Stand-alone type is rare distribution in *Streptomyces*; distance between *afsA* homolog and the receptor homolog is 63.2-kb in *S. mutabillis* (region 27), 90.9-kb in *S. purpurascens* (region 35), and 215-kb in *S. rimosus* subsp. *rimosus* (region 40). Here, we show other 4 example of stand-alone type (regions 5, 20, 28, 53) in Figure 5. Their possible receptor homologs could not be detected around 12 kb in their contigs. The absence of the receptor homologs may be due to the insufficient length of contigs. Extensive nucleotide sequencing on these strains may help to detect the partner receptor gene.

### Classification of signaling molecule receptors and pseudo-receptors

It is noteworthy that multiple receptor homologous genes are located on the regulatory-gene-island (Figures 3 and 4), and some of them (termed as ‘pseudo-receptors’) act as negative regulators for antibiotic production; for example, ScbR2 for coelimycin P-1 in *S. coelicolor* [33], BarB for virginiamycin in *Streptomyces virginiae* [64], and SrrB for lankacidin and lankamycin in *S. rochei* [46,49] (Table 1). Classification of ‘true’ signaling-molecule receptors and pseudo-receptors could be judged by their isoelectric point (pI) values. True receptors generally have acidic pI (∼5), whereas pseudo-receptors have basic pI values (∼9–11) [65]. We can also predict their functional difference through their genetic locations; the signaling-molecule receptor genes usually locate adjacent to the signaling-molecule biosynthetic genes, while the pseudo-receptor genes have no relationship with the biosynthetic genes [62]. In the case of the SRBs/SrrA system in an *S. rochei* linear plasmid pSLA2-L (Figure 3, region 46), a pseudo-receptor gene (*srrB*, *orf79*) is located vicinity to 4.8-kb from the signaling-molecule biosynthetic gene (*srrX*, *orf85*). This gene product SrrB is classified into pseudo-receptor through a mutational analysis [46,49]. In other representative examples listed in Table 1, *scbR2* is at 53.5-kb from *scbA* (Figure 3, region 10), and *barB* is at 1.2-kb from *barX* (Figure 3, region 51).

## Outlook

Thus, the signaling-molecule/receptor system is generally predictable due to their vicinal genetic location. However, alignment of eight well-characterized signaling-molecule receptor homologs (Figure 3) showed that their specific ligands are unable to predict in terms of their amino acid sequence homology. Extensive understanding of signaling-molecule/receptor system may lead to activate silent secondary metabolites through multiple gene inactivation or feeding of various signaling molecules.

## Summary

- Over 12,528 *Streptomyces* genome are sequenced to date, however, only 40 *Streptomyces* signaling molecules in 16 strains (0.13% of sequenced strains) were structure elucidated.
- The γ-butyrolatone-type (Type A), 2,3-disubstituted butenolide-type (Type B2), and furan-type (Type C) signaling molecules are biosynthesized by enzymes harboring an AfsA tandem repeat motif. Their *Streptomyces* signaling-molecule/receptor system is classified into two groups; regulatory-gene-island type and stand-alone type.
- The signaling-molecule/receptor system is generally predictable due to their vicinal genetic location, however, their specific ligands (signaling-molecules) are difficult to be predicted.
- Understanding of signaling-molecule/receptor system may lead to activate silent secondary metabolites through multiple gene inactivation or feeding of various signaling molecules.

## Open Access

Open access for this article is provided via our Subscribe to Open programme, made possible through the support of our subscribing institutions. Learn more here: https://portlandpress.com/pages/open_access_options_and_prices#conditional.

## Abbreviations

BGC: biosynthetic gene cluster
MMF: methylenomycin furan
